# A framework for assessing quality indicators in animal cancer registries: a case study

**DOI:** 10.3389/fvets.2026.1947787

**Published:** 2026-10-15

**Authors:** Marta Cresci, Francesca Cito, Antonio Petrini, Giovanni Di Teodoro, Marina Baffoni, Giuseppe Ru, Maria Ines Crescio

**Affiliations:** 1 Istituto Zooprofilattico Sperimentale dell'Abruzzo e del Molise (IZSAM), Teramo, Italy; 2 Istituto Zooprofilattico Sperimentale del Piemonte, Liguria e Valle D’Aosta (IZSPLV), Torino, Italy

**Keywords:** animal cancer registry, animal cancer reporting, data quality, one health, quality indicators, veterinary oncology

## Abstract

**Introduction:**

Animal cancer registries (ACRs) are becoming increasingly important in veterinary oncology and within the One Health framework. However, no standardized framework currently exists to assess their quality comprehensively. This study developed a structured quality assessment framework by adapting internationally recognized standards for human cancer registries with the aim of providing data managers of animal cancer registries, as well as those involved in establishing new ACRs, with a tool to support the evaluation of registry activities.

**Methods:**

A systematized literature review identified 16 quality indicators organized into six domains: registry organization, data sources, comparability, validity, timeliness, and completeness. As a proof of concept, the framework was applied to the Abruzzo (Italy) ACR.

**Results:**

All indicators were measurable using routinely collected registry data, demonstrating the feasibility of the proposed approach and enabling a comprehensive evaluation of registry performance across all six quality domains.

**Discussion:**

Although further validation in different registry configuration is warranted, this framework provides a practical foundation for standardized quality assessment, benchmarking, and the harmonization of animal cancer registries.

## 1 Introduction

According to the definition provided in “Cancer Epidemiology: Principles and Methods,” a cancer registry is an organization responsible for the systematic collection, storage, analysis, interpretation and reporting of data on subjects with cancer (1). Three types of human cancer registries are defined based on their source of information: hospital-based cancer registries, which collect all the cancer cases admitted to one or more hospitals, pathology-based cancer registries, which record histological data from one or more laboratories and population-based cancer registries, which retrieve all cancer cases occurring within the population at risk in a defined geographical area (2). Although hospital- and pathology-based cancer registries are primarily designed to support clinical care, population-based cancer registries are better suited to measure cancer occurrence within a defined population and provide the evidence needed to develop, implement, and evaluate strategies aimed at reducing the burden of cancer in the community (1, 2).

To gather as many cases as possible from the population at risk, multiple sources should be enrolled, mainly hospitals, laboratories and also death certificates. Data collection may be conducted through both active and passive approaches. Active collection requires human cancer registry staff to visit the sources of cases directly, such as hospitals (2). Integration of multiple data sources improves case ascertainment and enhances overall registry quality by improving the completeness of case ascertainment of the area under study (3).

Besides quantity, the importance of the quality of the data recorded in human cancer registries has long been emphasized. Since data quality depends on the procedures used to generate the information, it must also be demonstrated through appropriate validation checks (4). Therefore, the International Association of Cancer Registries (IACR) was formed in 1966 with the primary aim of cancer case collection and enabling comparability across registries (1). Additional initiatives arose afterwards with the same objective, including the European Network of Cancer Registries (ENCR) at the European level and the (AIRTum) at Italian level (5, 6).

The four dimensions identified to assess the quality of human cancer registries are (a) *comparability*, defined as the degree of adherence of the coding and classification procedures of the registry to international guidelines, (b) *validity*, referring to the proportion of cases with a given characteristic that truly possess it, (c) *completeness*, to ensure that all the cancer cases are captured by the registry, and (d) *timeliness*, defined as the rapidity of a human cancer registry to collect process and report complete data (7, 8).

Over time, veterinarians have developed a growing interest in establishing Animal Cancer Registries (ACRs) with approximately 25–30 initiatives described internationally over recent decades, ranging from local hospital- or pathology-based databases to regional population-based networks (9, 10). However, only a subset remains actively operational or routinely updated (9). Because approximately 34% of canine deaths are attributable to cancer (9) and companion animals can serve as valuable models for comparative oncology studies (11, 12), the establishment of standardized registries is increasingly recognized as a key priority within veterinary public health.

The Italian Framework, as summarized by Fonti and Millanta (9), has always been active in this field with several pathology- or hospital-based cancer and population-based animal cancer registries of Italy (9). Even if cancer case reporting is not mandatory for companion animals in Italy, the Italian Network of Laboratories for Veterinary Oncology (NILOV) was established in 2013 (13) with the intent to gather cancer diagnostic data from the Italian Veterinary Public Laboratories, veterinary schools, and canine cancer registries from different geographical areas into a single database. Although the establishment of this network does not include population data and thus represents a collection of pathological cases rather than a true cancer registry, it has successfully harmonized the collection of data across different organizations, establishing a shared, standardized dataset containing essential information.

Over time, several initiatives have been raised to promote the standardization of animal cancer registries (10, 14), notably the implementation of a comparative coding system for canine tumors (15), although further efforts are still needed in this area (16).

The primary objective of this study was to develop a standardized quality assessment framework for animal cancer registries, designed in analogy with the criteria and methodological standards adopted by human cancer registries for data managers of animal cancer registries, as well as those involved in establishing new ACRs. Rather than proposing a simple collection of indicators, the framework aims to provide a structured methodology for evaluating the overall quality of animal cancer registries across multiple domains, including organizational characteristics, data sources, comparability, validity, timeliness, and completeness. Unlike previous publications that focused on coding systems or individual quality measures, this study proposes the first structured framework integrating organizational and data-quality indicators into a unified assessment methodology for animal cancer registries.

To assess the applicability of the proposed framework, it was applied to the pathology-based Animal Cancer Registry of Abruzzo and Molise, maintained by the Istituto Zooprofilattico Sperimentale dell’Abruzzo e del Molise (IZSAM). The Abruzzo Animal Cancer Registry was used as a proof-of-concept case study to evaluate the feasibility of the framework and its ability to identify strengths and weaknesses, and opportunities for improvement in registry organization and data quality. By promoting a standardized approach to quality assessment, this study seeks to support future harmonization efforts and facilitate comparisons among animal cancer registries operating in different geographical and organizational configuration.

## 2 Materials and methods

### 2.1 Development of the animal cancer registry quality assessment framework

The animal cancer registry quality assessment framework was developed through a targeted systematized review of the scientific literature and institutional guidelines concerning both human cancer registry quality assessment and studies published about Italian animal cancer registries. A systematized review was executed following the typology of Grant and Booth. Although this methodology utilizes structured tools for search execution and descriptive synthesis, it pragmatically limits the overall scope by targeting specific databases and omitting standard quality or risk-of-bias appraisals (17). The description of the literature search was realized by recommendations from the Preferred Reporting Items for Systematic Reviews and Meta-Analyses (PRISMA) 2020 Statement and the PRISMA-S extension for reporting literature searches, when applicable, to ensure transparency (18, 19).

Literature searches were conducted using PubMed^1^ and Scopus.^2^ The search included articles published from database inception to the date of the last search, conducted in December 2025. Only publications written in English or Italian were considered eligible.

The search on bibliographic databases focused on publications describing quality assessment methods for human cancer registries and included the following keywords: (“cancer registry” OR “cancer registries”) AND (“data quality” OR “quality control”) AND (comparability OR validity OR timeliness OR completeness). An additional filter was applied to select documents related to human cancer registry quality that were classified as Review or Systematic Review articles. Studies describing the organization and operation of animal cancer registries to identify structural and operational characteristics relevant to veterinary oncology, particularly in the Italian context, were also retrieved by the following keywords: (“cancer registr*” OR “tumor registry” OR “tumour registry”) AND (“veterinary oncology” OR “animal cancer registry” OR “Veterinary Medicine”) AND (dog* OR canine* OR Dogs). The search strategy is reported in Supplementary Table 1. Duplicate records were identified and removed prior to title and abstract screening with Elsevier. Mendeley Reference Manager.^3^

For studies addressing human cancer registry quality assessment, inclusion criteria comprised publications describing general quality indicators applicable to human cancer registries, rather than indicators evaluated for a single specific registry. For Italian animal cancer registries, publications were included if they described the registry structure (including variables collected) as well as the data collection procedures implemented, within the Italian context. Studies reporting only epidemiological data, without providing information on registry structure and characteristics, were excluded.

The titles and abstracts of potentially relevant papers were screened by one researcher according to the predefined eligibility criteria.

On human cancer registry quality assessment, additional sources were retrieved from the grey literature search, conducted between March and December 2025, of the recognized national and international organization involved in cancer registration web pages, including the International Agency for Research on Cancer (IARC)^4^, the National Cancer Institute – Division of Cancer Control & Population Science (National Institutes of Health, NIH)^5^, the European Network of Cancer Registries (ENCR)^6^, and the Associazione Italiana Registro Tumori (AIRTum)^7^. The national and international organization web pages were consulted with iterative hand searching. Even in this case, only reports written in English or Italian were considered eligible.

Potential quality indicators identified in the selected documents were evaluated for their applicability to veterinary cancer registries according to three predefined selection criteria: (i) documented use in the quality assessment of human cancer registries, (ii) applicability to the organizational and epidemiological characteristics of veterinary cancer registries, and (iii) the availability of objective, reproducible, and routinely collectable data required for their calculation. Indicators requiring information not routinely available in veterinary surveillance systems or relying on unverifiable assumptions were excluded.

Following indicator selection, the framework was validated by the study group and adapted to the veterinary context while preserving, whenever possible, the conceptual principles adopted in human cancer registration.

Unlike existing quality indicator frameworks in healthcare, which are commonly validated through expert consensus methods (20, 21), the preliminary evaluation of the proposed framework was based on its application to a real-world animal cancer registry.

### 2.2 Abruzzo Animal Cancer Registry (ACR)

The pilot application of the proposed quality assessment framework was conducted using data from the Abruzzo ACR, established in 2014 and managed by the Istituto Zooprofilattico Sperimentale dell’Abruzzo e del Molise (IZSAM). The registry is pathology-based and currently includes neoplastic diagnoses generated by the IZSAM Histopathology Unit during routine diagnostic activities. Its primary purpose is to collect standardized information on animal tumors to monitor the occurrence and distribution of neoplastic diseases in the regional companion animal population (22).

Cancer case registration is currently based exclusively on passive data collection, meaning that it relies on cancer cases reported by veterinary practitioners, while no active case-finding procedures have been implemented. Cases are recorded when biological samples are voluntarily submitted by veterinary practitioners to the histopathology laboratory for diagnostic purposes. Since reporting animal cancer cases to the ACR is not mandatory, participation by veterinary practitioners is promoted through a regional funding scheme that covers the costs of histopathological examinations for samples submitted within the registry’s catchment area. This financial support was introduced following the inclusion of the Abruzzo Animal Cancer Registry within the Regional Prevention Plan, recognizing the registry as a One Health surveillance tool for monitoring cancer occurrence in companion animals and supporting environmental and public health surveillance.

The overall data flow of Abruzzo ACR is summarized in Figure 1.

**Figure 1:**
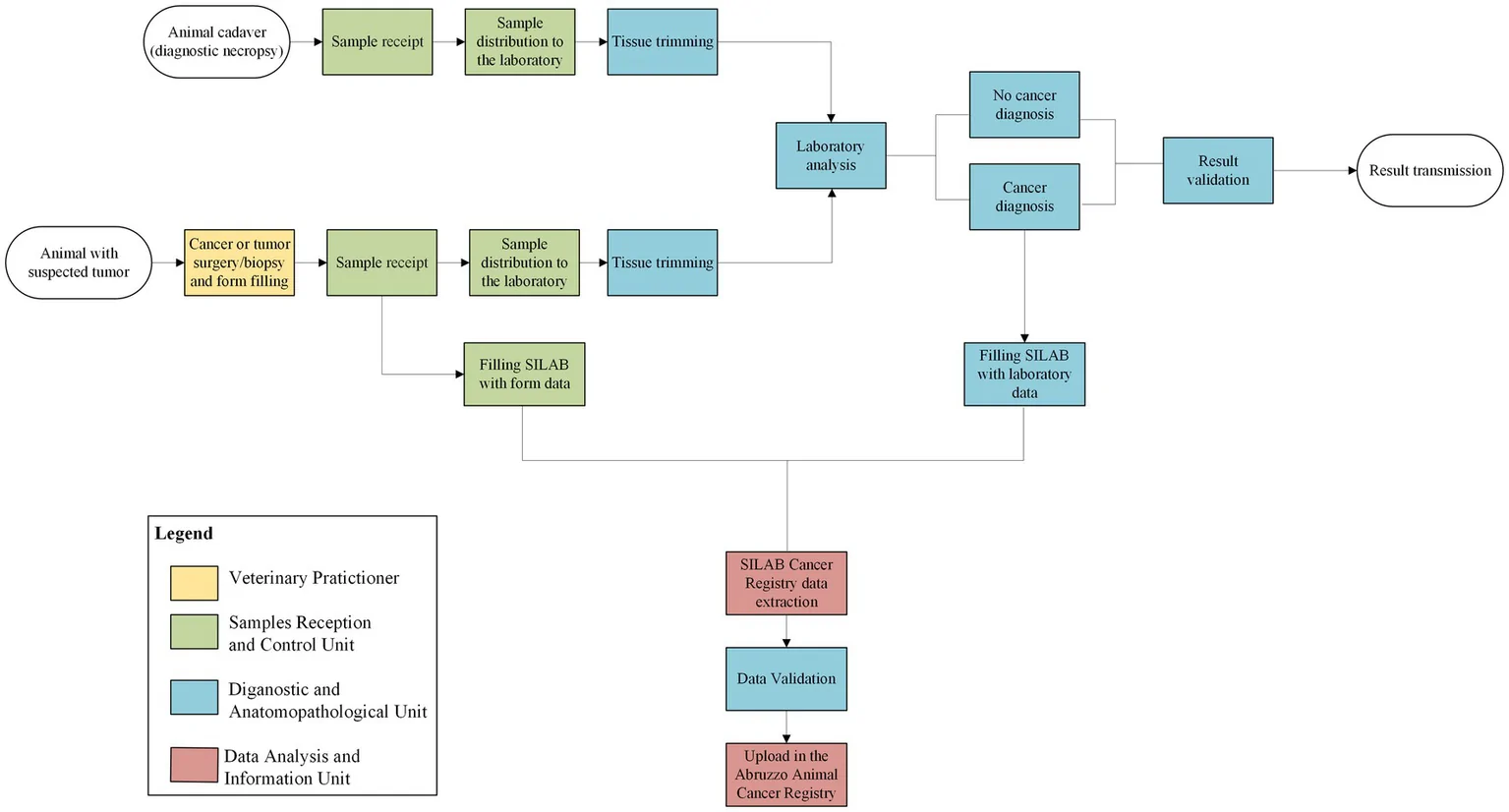
Summary of the Abruzzo ACR data flow. This figure illustrates how data are collected, recorded in IZSAM Laboratory Information Management System (SILAB), and processed in the Abruzzo Region Animal Cancer Registry. Since 2021, the ACR has also been collecting cases of cancer identified as incidental findings during necropsies performed by the IZSAM Diagnostics and Anatomopathological Unit, which were referred for investigation of other causes of death.

### 2.3 Data retrieval and preparation

For the proof-of-concept application, registry data were extracted from the IZSAM Laboratory Information Management System (SILAB). The study dataset included all samples submitted between 1st January 2020 and 31st December 2024 recorded within the “Cancer Registry” plan of the laboratory information system during sample accession. Data management, cleaning, and preparation procedures followed the workflow illustrated in Figure 2.

**Figure 2:**
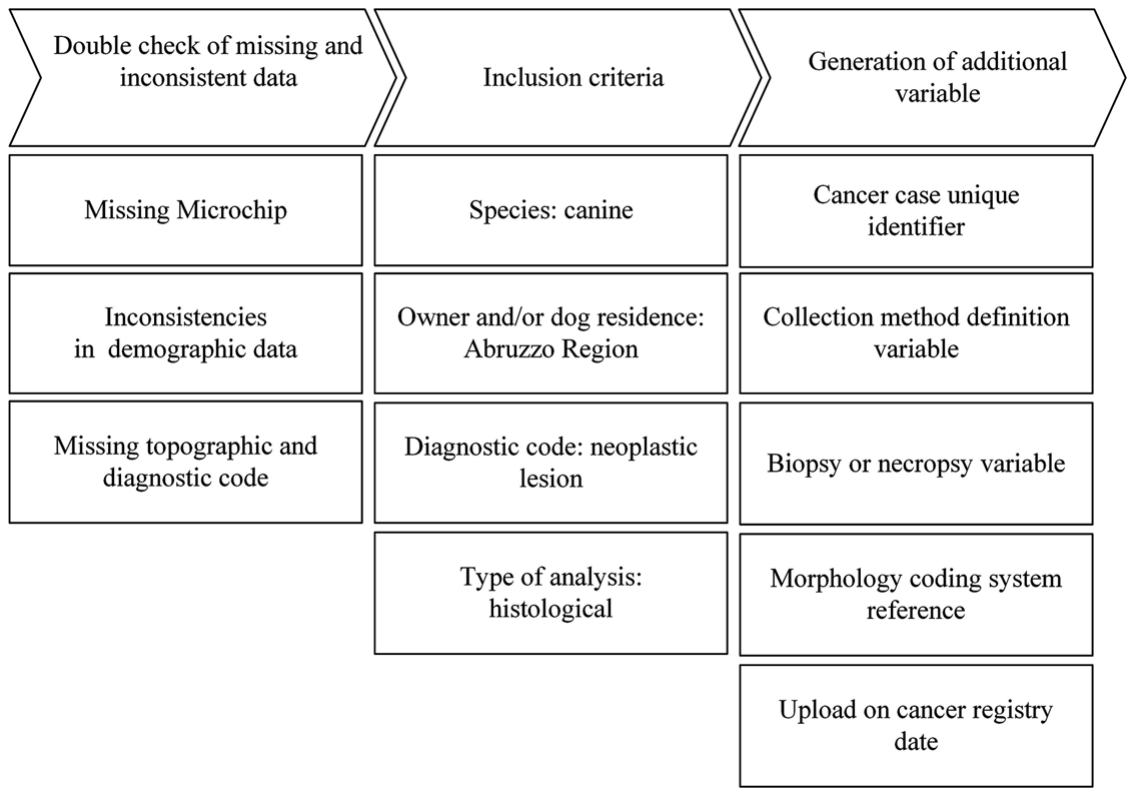
Summary of the Abruzzo ACR data management. The inclusion criterion regarding the type of analysis performed, namely the histological examination, was not included in the assessment of the sources of information for cases. The samples submitted and analyzed with histological examination represent only a portion of the total and would therefore provide an incomplete picture of the facilities involved.

For the analysis, only dogs were considered, excluding other species recorded in the Abruzzo Animal Cancer Registry. This choice was based on the Italian pet identification legislation, under which microchip implantation is mandatory for dogs, allowing individual animals to be uniquely identified when properly registered in SILAB.

Information on veterinary facilities operating in the Abruzzo Region was obtained from the Italian Companion Animal Information System (Sistema Informativo Nazionale degli Animali da Compagnia–SINAC) on 6 August, 2025. Veterinary practices are uniquely identified by a national unique code recorded in SINAC, which was used to harmonize and univocally identify reporting structures across datasets.

The performance of the selected indicators was evaluated by applying the framework to the Abruzzo ACR dataset. The results of indicators application to the Abruzzo ACR are summarized to evaluate the feasibility of the framework, describing the quality performance of the registry.

Statistical analyses were performed using Stata 19 (StataCorp. 2025. *Stata Statistical Software: Release 19*. College Station, TX: StataCorp LLC.) and Joinpoint Regression Software (23). The Joinpoint Regression Software was used to estimate the Average Annual Percent Change (AAPC) and the corresponding 95% confidence interval (CI). The AAPC is a statistical measure that summarizes the average annual percentage change over a specified period and is used to assess trends or changes in rates over time. In this analysis, the annual number of cases was used as the dependent variable and calendar year as the independent variable (24).

## 3 Results

### 3.1 Development of the quality assessment framework

The literature search was conducted to identify studies on both quality assessment methods for human cancer registries and Italian animal cancer registries. For the first review, a total of 566 records were identified. After removing duplicates and excluding articles that did not meet the predefined eligibility criteria (article type and language), 45 studies remained for screening. Following title and abstract assessment, 41 studies were excluded because they met the predefined exclusion criteria, resulting in 4 studies considered in the present review. A total of 7 reports from the grey literature were included in the present review following the eligibility assessment. A separate literature search was performed for studies on Italian animal cancer registries. A total of 28 records were identified, from which one duplicate was removed. After screening, 19 studies were excluded for not meeting the eligibility criteria, resulting in 8 studies being included in this paper.

The literature review flowchart is summarized in Figure 3.

**Figure 3:**
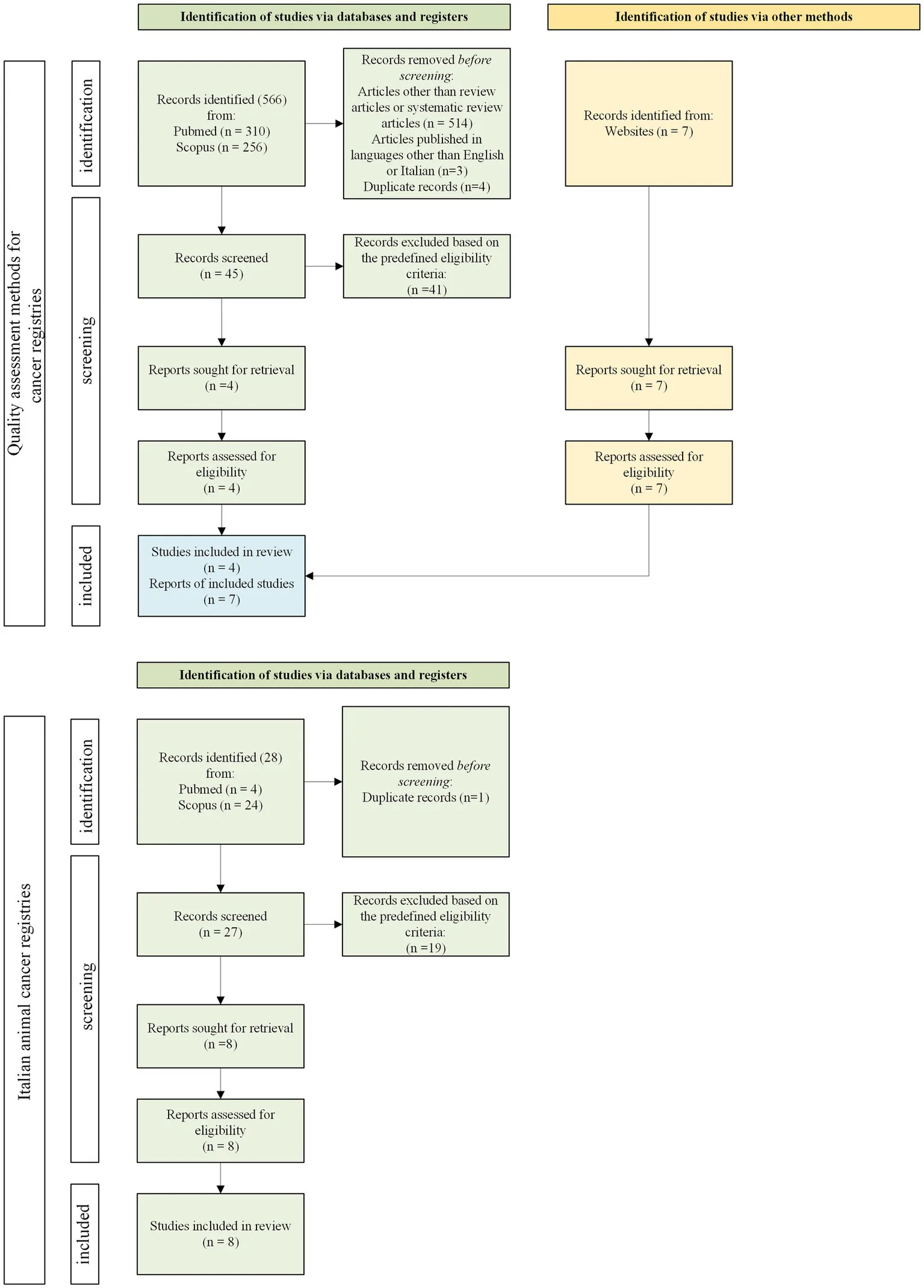
Literature review flow charts adapted and modified from the PRISMA 2020 guidelines for the identification and selection of quality assessment methods for cancer registries and for the identification and characterization of the structural and operational features of Italian animal cancer registries. The quality assessment methods for cancer registries flow chart includes records identified through searches of Scopus and PubMed, complemented by additional searches of grey literature sources. Records retrieved from Scopus and PubMed are shown in green, records identified through the grey literature search are shown in yellow, and the records included in the final qualitative assessment are shown in light blue. The Italian animal cancer registry flow chart includes records identified through Scopus and PubMed only, shown in green.

The framework was primarily based on internationally recognized methodological references for the evaluation of human cancer registries (1–3, 7, 8, 25–27), including the quality assessment models proposed by Parking and Bray, together with publications describing the organization and activities of Italian animal cancer registries (9, 13, 22, 28–32). A citation list of the studies included in the review is presented in Supplementary Tables S2, S3.

The literature review identified a set of quality assessment criteria commonly adopted in human cancer registries that could be adapted to the veterinary context.

Based on the predefined selection criteria, 16 quality indicators were retained and organized into six complementary quality domains: registry organization, data sources, comparability, validity, timeliness, and completeness. The complete framework, including the rationale, calculation method, and reference source for each indicator, is summarized in Table 1.

**Table 1:** Proposed quality assessment framework for animal cancer registries, including quality domains, indicators, objectives, calculation methods, and supporting references.

Domains	Indicator name	Aim	Description	Reference source
Registry organization (cancer data collection)	1.1 Proportion of passive data collection	Quantify cases collected through passive reporting	Number of cases identified via passive search/Total cases	(2, 3)
	1.2 Proportion of active data collection	Quantify cases collected through active reporting	Number of cases identified via active search/Total cases	(2, 3)
	1.3 Balance between active and passive case ascertainment	Assess the effort involved in the types of research carried out	Active cases/passive cases	(2, 3)
Cancer data source	2.1 Coverage of reporting facilities	Measure participation in ACR network	Number of veterinary facilities reporting suspected cases to the ACR in the jurisdiction Region/Total number of veterinary facilities in the jurisdiction region	(2)
Comparability	3.1 Use of standard coding systems	Assess compliance in the register of coding systems applied to reference standards	Define how many and which coding systems are applied	(7)
	3.2 Proportion of correctly prioritized incident dates	Assess standardized coding of incidence date	Proportion of cases in which the incident date is coded according to the recommended priority order of incident date definition out on the total number of cases	(7, 26)
	3.3 Primary tumors definition	Assess application of rules for defining the primary tumor	Define which rules for determining the primary tumor are applied	(7, 35)
	3.4 Proportion of primary tumors	Assess the proportion of the primary tumor recorded	Proportion of primary tumor / total cases	(7)
	3.5 Proportion of necropsy findings	Assess the proportion of tumors cases diagnosed at necropsy	Tumor cases identified only at necropsy	(7)
Validity	4.1 Missing animal identification/demographic information	Analysis of the missing information regarding the basic identification, demographic variables to evaluate the effectiveness of the data collection system.	Proportion of missing: Microchip Breed Sex Neuter status Date of birth Owner address Pet address	(7)
	4.2 Missing cancer information	Analysis of the missing information regarding the basic tumor data variables to evaluate the effectiveness of the data collection system.	Proportion of missing Date of diagnosis Method of diagnosis	(7)
	4.3 Proportion of cases with unknown primary site (PSU%)	Assess the Proportion of cases for which primary site is unknown.	The proportion of records with: an ICD9 topography code that includes 195 a Vet-ICD-O-canine-1 topography code equal to C80.9	(7)
Timeliness	5.1 Timeliness of registration of passively collected cases	Assess the timeliness of registration for passive cases.	Median of the difference between the registration date and the incident date of passive collected cases	(7)
	5.2 Timeliness of registration of active collected cases	Assess the timeliness of registration for active cases.	Median of the difference between the registration date and the incident date of active collected cases	(7)
	5.3 Overall registration timeliness	Assess the overall timeliness of registration.	Median of the difference between the registration date and the sampling date of all the cases	(7)
Completeness	6.1 Annual Percent Change (APC) / Average Annual Percent Change (AAPC)	Evaluate temporal stability of case trends, helping to identify potential changes in completeness over time.	Weighted average of the APCs (Annual Percent Change) resulted from the joinpoint model	(8, 25)

The first two domains, registry organization and data sources, were specifically introduced to address organizational aspects that are particularly relevant to veterinary cancer registries. Unlike most human cancer registries, veterinary registries frequently rely on voluntary participation by veterinary practices and may differ substantially in governance, funding mechanisms, reporting strategies, and geographical coverage. Assessing these organizational characteristics is therefore essential for correctly interpreting registry performance and representativeness.

The remaining four domains were adapted from internationally recognized quality assessment frameworks developed for human cancer registries. Comparability evaluates the degree of standardization achieved in case definition and coding practices, ensuring that registry data can be consistently interpreted and compared across settings. Validity focuses on the accuracy, internal consistency, and completeness of individual records, providing evidence of the reliability of the collected information. Timeliness assesses the efficiency of the registration process by measuring the interval between diagnosis and data availability, whereas completeness evaluates the ability of the registry to capture the target population. Because no independent reference source is currently available for estimating completeness in veterinary cancer registries, this domain was assessed through temporal trends in case registration using the Annual Average Percent Change (AAPC) calculated by the Joinpoint Regression Software.

Overall, the proposed framework integrates internationally recognized quality dimensions with indicators specifically designed to capture the unique organizational characteristics of veterinary cancer surveillance systems. Together, the 16 indicators provide a comprehensive approach for evaluating both registry performance and data quality, while maintaining applicability using routinely collected registry data.

### 3.2 Application of the framework to the Abruzzo Animal Cancer Registry

The selected indicators were applied to the Abruzzo ACR dataset by identifying the registry variables corresponding to those required for indicator calculation, according to the definitions and proportions described in Table 1. Indicator values were then calculated to assess the applicability of the proposed framework. Detailed results for each indicator are provided in the Supplementary material, while the main findings are summarized below and in Table 2 according to the six quality domains of the framework.

**Table 2:** Main results of the application of the quality assessment framework for animal cancer registries to Abruzzo Animal Cancer Registry during the period 2020–2024.

Dimensions	Indicator	Summary measure	Results over the study period (2020–2024)
1. Registry organization (cancer data collection)	1.1 Proportion of passive data collection	Median	100%
	1.2 Proportion of active data collection	Median	0%
	1.3 Balance between active and passive case ascertainment	-	Not applicable since no active data collection has been implemented
2. Cancer data source	2.1 Coverage of reporting facilities	Median (range)	23.38% (19.48–23.38%)
3. Comparability	3.1 Use of standard coding systems	-	All tumors were coded according standard coding system, in detail: 2,265 tumors were assigned an ICD-9 (International Classification of Diseases, Ninth Edition) topography code 1,828 tumors were coded according to ICD-O-3 (International Classification of Diseases for Oncology, Third Edition) morphology code 437 according to the Vet-ICD-O-Canine-1 (Veterinary International Classification of Diseases for Oncology Canine Tumors First Edition) morphology code
	3.2 Proportion of correctly prioritized incident dates	Median	100%
	3.3 Primary tumors definition	-	European Network of Cancer Registries (ENCR) primary tumor definition rules were not considered fully applicable.
	3.4 Proportion of primary tumors	Median (range)	Based on the practitioner definition: 96.58% (95.97–98.39%) Based on unique tumor identifier: 95,48% (93.35–97.71%)
	3.5 Proportion of necropsy findings	Median (range)	1.14% (0.73–2.04%)
4. Validity	4.1 Missing animal identification/demographic information	Median (range)	Microchip: 30% (20–34%) Breed: 1% (0–3%) Sex/neuter status: 6% (0–7%) Date of birth: 6% (1–16%) Owner address: 0% Pet address: 0%
	4.2 Missing cancer information	Median	Date of diagnosis: 0% Method of diagnosis: 0%
	4.3 Proportion of cases with unknown primary site (PSU%)	Median (range)	0%
5. Timeliness	5.1 Timeliness of registration of passively collected cases	Median (range)	8.61 months (2.4–23.7)
	5.2 Timeliness of registration of active collected cases	–	Not applicable since no active data collection has been implemented
	5.3 Overall registration timeliness	Median (range)	8.61 months (2.4–23.7)
6. Completeness	6.1 Annual Percent Change (APC) / Average Annual Percent Change (AAPC)	Annual Percent Change (APC) / Average Annual Percent Change (AAPC) (95% Confident intervals)	6.8381 (2.3595, 11.7014)

Regarding registry organization and data sources, a total of 2,265 tumors were registered between 2020 and 2024, with annual registrations increasing from 412 in 2020 to 525 in 2024. During the entire study period, all registered cases originated from passive case reporting, reflecting the current organization of the registry, while no active case-finding procedures were implemented (Supplementary Table S4). The number of veterinary facilities contributing to the registry ranged from 34 to 39 annually, corresponding to reporting coverage of 19.48 to 23.38% of all veterinary facilities operating in the Abruzzo Region (Supplementary Table S5).

Standardized coding systems were applied throughout the study period. All tumors were assigned an ICD-9 topography code. Morphological coding was performed in full using ICD-O-3 between 2020 and 2023. In 2024, 88 tumors were coded according to ICD-O-3 and 437 according to the Vet-ICD-O-Canine-1 morphology classification. (Supplementary Table S6).

The sampling date, acceptance date and validation date were available for all registered tumors during the study period and were used for incidence date prioritization. (Supplementary Table S7).

A tumor should be considered primary, in line with European Network of Cancer Registries, if it originates in a primary site or tissue and is not an extension, nor a recurrence, nor a metastasis, with multifocal tumors considered a single cancer. According to the referring practitioner, the proportion of tumors classified as primary ranged from 95.97 to 98.39% among cases with tumor type information available. Using the registry-generated unique tumor identifier, the proportion of primary tumors ranged from 93.35 to 97.71%. Necropsy-derived diagnoses represented between 0.73 and 2.04% of registered tumors, while biopsy specimens accounted for more than 97% of all cases in every study year (Supplementary Table S8–S10).

The proportion of missing demographic information decreased over the study period for most variables. Missing microchip identification ranged from 34% in 2020 to 20% in 2024. Missing breed information decreased from 3 to 0%, missing sex from 7% to less than 1%, and missing date of birth ranged from 41%, to 16% (median 6%). Owner address was available for all registered cases (Supplementary Table S11). The assessment of missing information for cancer-related variables showed that no missing values were present. The date of diagnosis was available for all cases throughout the study period, while the method of diagnosis was available for all registered tumors, as the Abruzzo ACR includes only histopathological examinations performed by the laboratory managing the registry (Supplementary Table S12). No tumors with an unknown primary site were recorded between 2020 and 2024 according to either the ICD-9 or the Vet-ICD-O-Canine-1 topography coding systems (Supplementary Table S13).

Registration timeliness for passively collected cases was assessed as the interval between the acceptance date and the registration date. The distribution of registration times for the study period is reported in Supplementary Figure S1. Indicators related to active registration timeliness were not calculated because no cases were collected through active case finding during the study period.

The annual number of registered tumors increased from 412 cases in 2020 to 525 cases in 2024. The temporal trend in case registration was assessed by estimating the Average Annual Percent Change (AAPC). Since the analysis covered a 5-year period and no joinpoints were included in the model according to the model selection criteria, the annual trend was constant across the entire interval, making the estimated APC and AAPC mathematically identical (6.84%, 95% CI: 2.36–11.70%; Supplementary Figure S2).

## 4 Discussion and conclusions

This study introduces a structured quality assessment framework for animal cancer registries, developed by adapting the principles and methodological standards widely adopted in human cancer registration systems. Unlike approaches based solely on individual quality indicators, the proposed framework integrates multiple dimensions of registry performance into a coherent assessment model encompassing registry organization, data sources, comparability, validity, timeliness, and completeness. Although it should be acknowledged that the literature review was conducted by a single researcher and potential selection bias may have been introduced at this stage, the quality indicators identified from the included literature were subsequently reviewed and validated by the study group.

As a limitation, no formal risk-of-bias assessment of the included studies was performed, consistent with the systematized approach adopted in this study. However, the evidence base also included authoritative sources from IARC, ENCR, and AIRTUM, which undergo internal review and consensus processes before publication.

The resulting framework was designed for data managers of animal cancer registries, as well as those involved in establishing new ACRs, to provide a structured assessment of registry performance, identify strengths and weaknesses in registry operations, and support future standardization and benchmarking initiatives in veterinary cancer registration.

The application of the framework to the Abruzzo ACR should be considered a proof-of-concept demonstrating its feasibility rather than a formal validation, providing a preliminary assessment of the potential usefulness of selected indicators for evaluating the quality of animal cancer registries. Further evaluation across multiple animal cancer registries, supported by structured expert consensus, will be necessary to establish its validity and generalizability. However, beyond the evaluation of a single registry, the framework is intended to provide a common methodological basis for the assessment and comparison of animal cancer registries operating in different settings. The findings provide an initial basis for further methodological development.

The application of the framework to the Abruzzo ACR showed that the registry performed well across most of the proposed quality domains. Standardized coding systems were consistently applied, demographic and cancer-related data completeness improved over time, the proportion of primary tumors remained consistently high, and tumors with unknown primary site were not recorded. Registration timeliness remained stable throughout the study period. The assessment also identified areas requiring further development, including limited participation by veterinary facilities in the reporting network, exclusive reliance on passive case ascertainment, and the need for full implementation of internationally recognized rules for multiple primary tumors.

The evaluation of registry organization and data sources is particularly important because it provides information on the effort required to collect cancer data and maintain surveillance activities over time. Cancer data may be collected through passive or active approaches, depending on the available resources and organizational structure (2). In Italy, where reporting of animal cancer cases is not mandatory, passive surveillance largely relies on the willingness of veterinary practitioners to submit cases. For this reason, several initiatives have been implemented to encourage participation, including free-of-charge histopathological examinations (9, 22, 28, 31). Active case finding, on the other hand, requires dedicated personnel to access and review clinical databases, laboratory information systems, or veterinary management software and often involves substantial efforts to standardize retrospectively collected information. Evaluating these aspects is essential not only to understand registry performance but also to estimate the resources required to ensure sustainable operation and continuous engagement of reporting facilities (2).

Passive reporting for the Abruzzo ACR also implies that histopathological diagnoses are performed exclusively by the IZSAM laboratory. While this guarantees high diagnostic consistency and strict adherence to standardized coding systems, it inevitably affects case completeness, as tumor cases diagnosed by private external laboratories or clinics referring outside the regional borders are not captured. In the absence of a dedicated survey, the extent of potential case under-ascertainment cannot be directly quantified, as companion animal cancer reporting is non-mandatory in Italy and lacks an independent gold-standard administrative database. The availability of free histopathological examinations may nevertheless encourage case submission from veterinary practitioners however, samples may also be submitted to private laboratories for other reasons, such as convenience, turnaround time, or established professional relationships. In addition, Abruzzo borders four regions (Umbria, Lazio, Marche, and Molise). With the exception of Molise Region, where the animal cancer registry is under IZSAM management, cases may be missed because veterinary practices located in neighboring areas may serve overlapping animal populations. Future studies could investigate these aspects by linking cancer registry data with other available sources, such as the national canine registry and veterinary prescription databases, which may provide complementary information for assessing case ascertainment and, potentially, completeness.

The assessment of data sources also provides insight into the territorial coverage of reporting facilities and the degree of participation of veterinary practitioners. Adequate geographic coverage is fundamental to ensure that reported cases are representative of the monitored population. In the present case study, approximately one-fifth of veterinary facilities in the Abruzzo Region contributed to the registry throughout the study period. Increasing the number of reporting facilities remains an important objective to improve the representativeness and long-term sustainability of the registry. In addition, the type of facilities contributing data should be considered, as oncology consultations and surgical procedures are more frequently performed in veterinary clinics and referral centers than in smaller practices, since, under Italian law, a veterinary clinic is required, as a minimum structural standard, to have an operating room and the capacity to accommodate animal inpatients overnight (33). These considerations may support future strategies aimed at improving case ascertainment and strengthening collaboration between registries and veterinary professionals.

Comparability reflects the extent to which registry data can be reliably compared across different populations and settings (7). A major challenge in veterinary oncology is the absence of internationally accepted standards equivalent to those available for human cancer registries. In the present case study, comparability was assessed with reference to the coding systems adopted for reporting to CEROVEC, including ICD-9 and, more recently, Vet-ICD-O-canine-1 for tumor morphology (15). The gradual adoption of Vet-ICD-O-Canine-1 for morphology coding represents an important step towards greater harmonization of veterinary cancer registration. In addition to coding consistency, comparability requires standardized criteria for defining incidence date and identifying primary tumors, both of which are essential for producing robust epidemiological estimates and ensuring consistency among registries.

Another aspect relevant to comparability is the contribution of necropsy-derived diagnoses. According to IARC recommendations, cancers identified during necropsy may influence the estimation of cancer occurrence. This issue is particularly relevant in veterinary oncology, where systematic screening programs are generally absent, and cancer registration is voluntary. Consequently, the proportion of cancers identified only at necropsy should be monitored, especially when interpreting temporal variations in the frequency of specific tumor types (7).

Validity indicators assess the reliability of recorded information and the effectiveness of data collection and registration procedures. Their evaluation allows the identification of inconsistencies, potential sources of error, and weaknesses in registry operations, thereby providing insight into the robustness of the information generated by the registry. While in human cancer registration validity strictly reflects diagnostic accuracy and the proportion of cases with a given characteristic that truly possess it—typically verified through re-abstracting audits, external source concordance, or diagnostic confirmation procedures—its operational definition often requires adaptation in veterinary epidemiology. The decision to include missing information analysis among the validity indicators was based on its applicability to the veterinary context and to the data sources available in the registry. In veterinary cancer registration, tumor diagnoses are often based on histopathological examinations performed by the same laboratory responsible for the registry, limiting the possibility of independent re-abstraction or validation against multiple external sources. Similarly, death-certificate-only (DCO) registration is not applicable, as equivalent standardized mortality documentation is not available in veterinary medicine. In contrast, missing information analysis can be directly performed using the information routinely recorded in the registry and can provide an indication of potential weaknesses in data collection and registration procedures. Internal consistency assessment may also be relevant; however, its application in animal cancer registries requires dedicated investigation to define and validate appropriate criteria based on the evidence currently available. Future developments should therefore aim to incorporate targeted cross-checks with secondary data sources (such as regional companion animal databases or veterinary clinical records) to further strengthen the formal assessment of diagnostic and demographic accuracy.

In the present case study, the application of the framework to the Abruzzo ACR also documented a progressive reduction in missing demographic information over time, while missing cancer-related variables remained negligible throughout the study period. This pattern suggests progressive refinement of data collection procedures and increasing adherence of reporting laboratories to the registry data requirements.

Timeliness represents another key quality dimension, as it reflects the speed with which data are collected, processed and made available for analysis. Because reporting dynamics may differ substantially between passive and active surveillance systems, separate evaluation of these components may provide a more accurate assessment of registry performance. While passive reporting can occur continuously throughout the year, active case-finding activities are often conducted periodically according to the availability of personnel and financial resources.

Completeness describes the extent to which a registry captures all cancer cases occurring within the target population and serves as a key quality indicator that determines the reliability of cancer statistics. The approaches proposed by Parkin and Bray (8) for assessing cancer registry completeness were considered in relation to their applicability to the current veterinary context. These include historic data methods, such as the assessment of incidence stability over time, comparison of incidence rates across populations, and evaluation of age-specific incidence patterns, as well as approaches based on mortality-to-incidence ratios, multiple notification sources, and histological verification of diagnosis. Quantitative methods, including independent case ascertainment, capture–recapture, and death certificate-based approaches, may provide more direct assessments of completeness when appropriate independent data sources are available. However, their applicability in veterinary medicine is currently limited by several methodological and practical constraints, including the lack of independent and standardized data sources, incomplete mortality information, difficulties in linking historical records, heterogeneous data collection practices, and the limited availability of reliable population denominators. In addition, approaches such as independent case ascertainment would require systematic active case-finding, dedicated personnel, coordination among multiple stakeholders, and substantial additional resources.

These limitations are particularly relevant to the Abruzzo Animal Cancer Registry. Unlike population-based human cancer registries, the Abruzzo ACR currently operates in a context in which the population at risk is not defined through a comprehensive and standardized population registry. Animal populations are dynamic, identification practices may vary, and veterinary care is largely provided through a private and decentralized system. Consequently, defining a reliable denominator for the calculation of population-based incidence rates is challenging. Similarly, cancer cases are not systematically identified through independent active case-finding, and mortality data are incomplete. These characteristics currently limit the reliability and feasibility of conventional quantitative approaches to completeness assessment.

Among the available approaches, the average annual percentage change (AAPC) estimated through Joinpoint regression Software was considered particularly suitable for animal cancer registries (34).

In this context, the analysis of temporal trends in the number of registered cases provides a useful proxy of registry development and source engagement. A sustained increase in registered cases over time may reflect improved participation of reporting facilities and progressive strengthening of surveillance and registration activities. However, an increase in registered cases cannot, by itself, be interpreted as evidence of improved case ascertainment, as it may also result from changes in disease occurrence, changes in the population under surveillance, diagnostic practices, case definitions, reporting behavior, or surveillance intensity. Therefore, the interpretation of temporal trends as an indication of improved ascertainment assumes that these factors remain sufficiently stable over time, or that relevant changes can be accounted for. Biologically, an annual increase of almost 7% over a five-year period is far too rapid to represent a true shift in underlying population tumor incidence, which tends to change slowly. It should therefore be interpreted cautiously, as it may reflect changes in ascertainment and registration rather than a corresponding biological increase in tumor incidence. Instead, it is far more consistent with surveillance network consolidation. It should be emphasized that the AAPC is not intended as a direct measure of registry completeness. Rather, within the context of veterinary cancer registries, it should be interpreted as an indirect indicator of registry maturation, reflecting the progressive stabilization of reporting practices and engagement of participating facilities. Consequently, changes in AAPC should always be interpreted together with information on registry organization and data sources. In the Abruzzo ACR, the progressive increase in the annual number of registered tumors were accompanied by stable reporting coverage and improvements in data completeness, supporting the interpretation that the observed trend is more likely to reflect registry maturation than changes in disease occurrence.

APC/AAPC was therefore selected as the most feasible indicator in the current setting, as it can be estimated using routinely available registry data without requiring additional independent data sources or active case-finding procedures. This choice reflects the specific characteristics and current limitations of veterinary cancer registration and should be regarded as a pragmatic component of the proposed framework, rather than as an exclusive approach to assessing registry completeness. As animal cancer registries become more widely established and data collection becomes increasingly standardized and integrated, complementary approaches could be progressively incorporated to provide a more comprehensive assessment of completeness.

In the future, comparison of temporal trend patterns across animal cancer registries covering comparable veterinary populations could provide an additional indirect means of assessing data completeness. For instance, as demonstrated by Cancer Incidence in Five Continents (8), establishing expected incidence ranges based on data from comparable populations may help distinguish geographical or population-specific differences from patterns potentially related to inconsistencies in registration. The increasing comprehensiveness of animal cancer and population registries may provide standardized and comparable data, thereby facilitating the interpretation of differences in case numbers and incidence rates across populations.

This study has some limitations. First, the framework was applied to a single animal cancer registry, and therefore its performance has not been evaluated under different organizational and epidemiological situations. In addition, some indicators, particularly those related to active case ascertainment, could not be assessed because the Abruzzo ACR currently relies exclusively on passive reporting. Finally, although the proposed indicators were selected through a structured review of the available literature, their relative importance has not yet been formally weighted through expert consensus.

The proposed framework should be viewed as an evolving methodology. Its application to additional animal cancer registries will provide evidence of its generalizability, while future international expert consensus may help prioritize and weight the proposed indicators according to their relative contribution to overall registry quality, rather than redefining the framework itself. In this context, consensus-based approaches, such as Delphi panels, involving veterinary oncologists, epidemiologists, and health data managers, could be used to confirm the relevance of the selected indicators and to define weighted scoring systems capable of generating an overall registry quality score. Such developments would facilitate benchmarking among registries and contribute to the establishment of harmonized quality standards for animal cancer registries at both national and international levels.

In conclusion, the proposed framework represents an important first step toward the standardization of quality assessment procedures for animal cancer registries. By providing a structured methodology for evaluating registry organization, data quality, and surveillance performance, it may support future harmonization efforts and promote more reliable and comparable veterinary oncology data within the One Health framework.

## Data Availability

The datasets presented in this article are not publicly available, as they are maintained by the Abruzzo Region Animal Cancer Registry. Requests to access these datasets should be directed to GT (g.diteodoro@izs.it), Istituto Zooprofilattico Sperimentale dell’Abruzzo e del Molise (IZSAM).

## Footnotes

1
https://pubmed.ncbi.nlm.nih.gov/

2
https://www.scopus.com/pages/home#basic

3
https://www.mendeley.com/reference-manager/

4
https://www.iarc.who.int/

5
https://cancercontrol.cancer.gov/

6
https://www.encr.eu/

7
https://www.registri-tumori.it/cms/
