# Supplementary material for: A framework for assessing quality indicators in animal cancer registries: a case study
Source: Front Vet Sci. 2026 Oct 15;13:1947787. doi: 10.3389/fvets.2026.1947787 (PMC13717246; doi:10.3389/fvets.2026.1947787)
Supplement: Supplementary file 1 [file Table_1.DOCX]

Supplementary Material

**Supplementary Table S1. Search Strategy**

| **Database** | **Search details on cancer registry quality assessment** | **Search details on Italian animal cancer registries** |
| --- | --- | --- |
| **Pubmed** | ("cancer registry" OR "cancer registries") AND ("data quality" OR "quality control") AND (comparability OR validity OR timeliness OR completeness) | ("cancer registr*" OR "tumor registry" OR "tumour registry") AND ("veterinary oncology" OR "animal cancer registry" OR "Veterinary Medicine") AND (dog* OR canine* OR Dogs) |
| **Scopus** | TITLE-ABS-KEY ( ( "cancer registry" OR "cancer registries" ) AND ( "data quality" OR "quality control" ) AND ( comparability OR validity OR timeliness OR completeness ) ) | TITLE-ABS-KEY ( ( "cancer registr*" OR "tumour registry" OR "tumor registry" ) AND ( "veterinary oncology" OR "animal cancer registry" OR "canine cancer registry" ) AND ( dog OR dogs OR canine OR canines OR veterinary ) ) |

**Supplementary Table S2. References included in the non-systematic literature review on quality assessment methods for human cancer registries**

| **Reference** | **Author(s)** | **Year** | **Title** |
| --- | --- | --- | --- |
| 1 | Dos Santos Silva | 1999 | Cancer Epidemiology: Principles and Methods |
| 2 | Bray F, Znaor A, Cueva P, Korir A, Swaminathan R, Ullrich A, Wang SA, Parkin DM | 2014 | PLANNING AND DEVELOPING POPULATION-BASED CANCER REGISTRATION IN LOW-AND MIDDLE-INCOME SETTINGS |
| 3 | Ferretti S, Giacomin A, Gruppo di lavoro Associazione Italiana Registri Tumori (AIRTUM) | 2007 | Manuale di Tecniche di registrazione dei Tumori 2008 |
| 7 | Bray F, Parkin DM | 2009 | Evaluation of data quality in the cancer registry: Principles and methods. Part I: Comparability, validity and timeliness. |
| 8 | Parkin DM, Bray F | 2009 | Evaluation of data quality in the cancer registry: Principles and methods Part II. Completeness. |
| ~~24~~25 | National Cancer Institute, Division of Cancer Control and Population Sciences | - | Average Annual Percent Change (AAPC) and Confidence Interval — Joinpoint Help System |
| ~~25~~26 | European Network of Cancer Registries (ENCR) | 2022 | Coding Incidence Date |
| ~~26~~27 | Sousa MP, Monjardino T, Santos CC, Lara L, Bento MJ. | 2025 | Completeness Evaluation of Adult-Population-Based Cancer Registries: A Systematic Review |

**Supplementary Table S3. References included in the non-systematic literature review on** **Italian animal cancer registries**

| **Reference** | **Author(s)** | **Year** | **Title** |
| --- | --- | --- | --- |
| 9 | Fonti N, Millanta F. | 2025 | Cancer registration in dogs and cats: A narrative review of history, current status, and standardization efforts. |
| 13 | Crescio MI, Ru G, Aresu L, Bozzetta E, Cancedda MG, Capello K, Castagnaro M, Carnio A, Cocumelli C, Degli Uberti B, Eleni C, Foiani G, Fonti N, Gibelli LR, Maniscalco L, Manuali E, Moccia V, Paciello O, Petrella A, Petrini A, Poli A, Puleio R, Razzuoli E, Scaramozzino P, Varello K, Vascellari M, Zappulli V, Ferrari A, On Behalf Of Nilov. | 2022 | The Italian Network of Laboratories for Veterinary Oncology (NILOV) 2.0: Improving Knowledge on Canine Tumours. |
| ~~21~~22 | Di Teodoro G, Cito F, Salini R, Baffoni M, Defourny SVP, Cocco A, D’Alterio N, Palmieri C. | 2024 | Pathology-Based Animal Cancer Registry of Abruzzo and Molise Regions (Central Italy): A Ten-Year Retrospective Study (2014–2023). |
| ~~27~~28 | Baioni E, Scanziani E, Vincenti MC, Leschiera M, Bozzetta E, Pezzolato M, Desiato R, Bertolini S, Maurella C, Ru G. | 2017 | Estimating canine cancer incidence: findings from a population-based tumour registry in northwestern Italy. |
| ~~28~~29 | Vascellari M, Baioni E, Ru G, Carminato A, Mutinelli F. | 2009 | Animal tumour registry of two provinces in northern Italy: incidence of spontaneous tumours in dogs and cats. |
| ~~29~~30 | Merlo DF, Rossi L, Pellegrino C, Ceppi M, Cardellino U, Capurro C, Ratto A, Sambucco PL, Sestito V, Tanara G, Bocchini V. | 2008 | . Cancer Incidence in Pet Dogs: Findings of the Animal Tumor Registry of Genoa, Italy. |
| ~~30~~31 | Manuali E, Morgante RA, Maresca C, Leonardi L, Purificato I, Giaimo MD, Giovannini G. | 2019 | A web-based tumor registration system for a regional Canine Cancer Registry in Umbria, central Italy. |
| ~~31~~32 | De Biase D, Baldassarre V, Piegari G, Rosato G, Caputo V, Pompameo M, Sarnelli P, Russo V, D’Angelo D, Papparella S, Paciello O. | 2023 | ANIMAL SENTINELS AND CANCER REGISTRIES: STATE OF THE ART AND NEW PERSPECTIVES. |

**Supplementary Table S4. Data collection on cancer cases of Abruzzo Animal Cancer Registry (ACR) for the period 2020–2024**

| **Year** | **Total number of tumors (n)** | **Total number of cases identified via passive search (n)** | **Proportion of passive data collection (%)** | **Total number of cases identified via active search (n)** | **Proportion of active data collection (%)** |
| --- | --- | --- | --- | --- | --- |
| **2020** | 412 | 412 | 100 | 0 | 0 |
| **2021** | 406 | 406 | 100 | 0 | 0 |
| **2022** | 442 | 442 | 100 | 0 | 0 |
| **2023** | 480 | 480 | 100 | 0 | 0 |
| **2024** | 525 | 525 | 100 | 0 | 0 |

**Supplementary Table S5. Source of information on cancer cases of Abruzzo ACR for the period 2020–2024. Data on total number of facilities in the Abruzzo region were obtained from the Italian pet information system (Sistema Informativo Nazionale degli Animali da Compagnia - SINAC) as of 6th of August, 2025.**

| **Year** | **Total number of facilities in Abruzzo Region (n)** | **Reporting facilities (n)** | **Coverage of reporting facilities (%)** |
| --- | --- | --- | --- |
| **2020** | 154 | 34 | 21.43 |
| **2021** | 154 | 36 | 19.48 |
| **2022** | 154 | 39 | 23.38 |
| **2023** | 154 | 37 | 23.38 |
| **2024** | 154 | 39 | 23.38 |

**Supplementary Table S6. Compliance with standard coding systems among cancer cases in the Abruzzo Cancer Registry (ACR), 2020–2024**

| **Year** | **Total number of tumors (n)** | **Number of tumors with compliance with the standard topography coding system in the Abruzzo ACR (n)** | **Number of tumors with compliance with the standard morphological coding system in the Abruzzo ACR (n)** | |
| --- | --- | --- | --- | --- |
|  |  | **ICD9** | **ICD-O-3** | **Vet-ICD-O-Canine-1** |
| **2020** | 412 | 412 | 412 | 0 |
| **2021** | 406 | 406 | 406 | 0 |
| **2022** | 442 | 442 | 442 | 0 |
| **2023** | 480 | 480 | 480 | 0 |
| **2024** | 525 | 525 | 88 | 437 |

**Supplementary Table S7. availability of the date sources used for incidence date prioritization among cancer cases in the Abruzzo Cancer Registry (ACR), 2020–2024**

| **Variable** | **2020** | | | **2021** | | | **2022** | | | **2023** | | | **2024** | | |
| --- | --- | --- | --- | --- | --- | --- | --- | --- | --- | --- | --- | --- | --- | --- | --- |
|  | **Total Samples** | **Missing (n)** | **Missing (%)** | **Total Samples** | **Missing (n)** | **Missing (%)** | **Total Samples** | **Missing (n)** | **Missing (%)** | **Total Samples** | **Missing (n)** | **Missing (%)** | **Total Samples** | **Missing (n)** | **Missing (%)** |
| **Sampling date** | 412 | 0 | 0 | 406 | 0 | 0 | 442 | 0 | 0 | 480 | 0 | 0 | 525 | 0 | 0 |
| **Acceptance date** | 412 | 0 | 0 | 406 | 0 | 0 | 442 | 0 | 0 | 480 | 0 | 0 | 525 | 0 | 0 |
| **Validation date** | 412 | 0 | 0 | 406 | 0 | 0 | 442 | 0 | 0 | 480 | 0 | 0 | 525 | 0 | 0 |

**Supplementary Table S8. Proportion of primary tumors recorded in the Abruzzo Animal Cancer Registry (ACR) for the period 2020–2024 estimated based on the definition provided by the referring practitioner, when available.**

| **Year** | **Total number of tumors with tumor type information available (n)** | **Primary Tumor (n)** | **Proportion of primary tumors (%)** |
| --- | --- | --- | --- |
| **2020** | 215 | 207 | 96.28 |
| **2021** | 280 | 275 | 98.21 |
| **2022** | 273 | 262 | 95.97 |
| **2023** | 322 | 311 | 96.58 |
| **2024** | 372 | 366 | 98.39 |

**Supplementary Table S9. Proportion of primary tumors recorded in the Abruzzo Animal Cancer Registry (ACR) during the period 2020–2024, estimated using the unique tumor identifier variable generated from cancer registry data.**

| **Year** | **Total number of tumors (n)** | **Total number of primary tumor (n)** | **Proportion of primary tumors (%)** |
| --- | --- | --- | --- |
| **2020** | 412 | 389 | 94.42 |
| **2021** | 406 | 379 | 93.35 |
| **2022** | 442 | 422 | 95.48 |
| **2023** | 480 | 468 | 97.50 |
| **2024** | 525 | 513 | 97.71 |

**Supplementary Table S10. Proportion of necropsy findings recorded in the Abruzzo Animal Cancer Registry (ACR) during the period 2020–2024.**

| **Year** | **Total number of tumors (n)** | **Total number of bioptic sample (n)** | **Proportion of bioptic sample (%)** | **Total number of necropsy sample (n)** | **Total number of**  **necropsy sample (%)** |
| --- | --- | --- | --- | --- | --- |
| **2020** | 412 | 409 | 99.27 | 3 | 0.73 |
| **2021** | 406 | 402 | 99.01 | 4 | 0.99 |
| **2022** | 442 | 433 | 97.96 | 9 | 2.04 |
| **2023** | 480 | 473 | 98.54 | 7 | 1.46 |
| **2024** | 525 | 519 | 98.86 | 6 | 1.14 |

**Supplementary Table S11. Missing animal identification/demographic information in the Abruzzo Animal Cancer Registry (ACR) during the period 2020–2024.**

| **Variable** | **2020** | | | **2021** | | | **2022** | | | **2023** | | | **2024** | | |
| --- | --- | --- | --- | --- | --- | --- | --- | --- | --- | --- | --- | --- | --- | --- | --- |
|  | **Total Samples** | **Missing (n)** | **Missing (%)** | **Total Samples** | **Missing (n)** | **Missing (%)** | **Total Samples** | **Missing (n)** | **Missing (%)** | **Total Samples** | **Missing (n)** | **Missing (%)** | **Total Samples** | **Missing (n)** | **Missing (%)** |
| **Microchip** | 412 | 142 | 34% | 406 | 124 | 31% | 442 | 131 | 30% | 480 | 104 | 22% | 525 | 107 | 20% |
| **Breed** | 412 | 14 | 3% | 406 | 3 | 1% | 442 | 7 | 2% | 480 | 4 | 1% | 525 | 0 | 0% |
| **Sex** | 412 | 29 | 7% | 406 | 37 | 9% | 442 | 28 | 6% | 480 | 11 | 2% | 525 | 1 | 0% |
| **Date of birth** | 412 | 18 | 4% | 406 | 63 | 16% | 442 | 72 | 16% | 480 | 5 | 1% | 525 | 3 | 1% |
| **Owner Address** | 412 | 0 | 0% | 406 | 0 | 0% | 442 | 0 | 0% | 480 | 0 | 0% | 525 | 0 | 0% |

**Supplementary Table S12. Missing cancer information in the Abruzzo Animal Cancer Registry (ACR) during the period 2020–2024.**

| **Variable** | **2020** | | | **2021** | | | **2022** | | | **2023** | | | **2024** | | |
| --- | --- | --- | --- | --- | --- | --- | --- | --- | --- | --- | --- | --- | --- | --- | --- |
|  | **Total Samples** | **Missing (n)** | **Missing (%)** | **Total Samples** | **Missing (n)** | **Missing (%)** | **Total Samples** | **Missing (n)** | **Missing (%)** | **Total Samples** | **Missing (n)** | **Missing (%)** | **Total Samples** | **Missing (n)** | **Missing (%)** |
| **Date of diagnosis** | 412 | 0 | 0% | 406 | 0 | 0% | 0 | 0 | 0% | 480 | 0 | 0% | 525 | 0 | 0% |
| **Method of diagnosis** | 412 | 0 | 0% | 406 | 0 | 1% | 0 | 0 | 0 | 480 | 0 | 0% | 525 | 0 | 0% |

**Supplementary Table S13. Proportion of cases with unknown primary site (PSU%) in the Abruzzo Animal Cancer Registry (ACR) during the period 2020–2024.**

| **Year** | **Total number of tumors (n)** | **Tumors with unknown primary site (n)** | **Proportion of unknown primary site (PSU%)** | |
| --- | --- | --- | --- | --- |
|  |  |  | ICD9 topography code that includes 195 | Vet-ICD-O-canine-1 topography code equal to C80.9 |
| **2020** | 412 | 0 | 0 | 0 |
| **2021** | 406 | 0 | 0 | 0 |
| **2022** | 442 | 0 | 0 | 0 |
| **2023** | 480 | 0 | 0 | 0 |
| **2024** | 525 | 0 | 0 | 0 |

**Supplementary Figure S1.** Timeliness of registration of passively collected cases in the Abruzzo Region, measured as the time between acceptance date (the date of acceptance was used as a proxy for the incident date) and registration in the Animal Cancer Registry (ACR) date (expressed in months), 2020–2024 (corresponding to overall registration timeliness as passive collection was the only method applied)

**Supplementary Figure S2.** Annual Percent Change (APC) for the Abruzzo Animal Cancer Registry (ACR) during the period 2020–2024.
